# A Rare Case of Hibernoma Occasionally Identified by 18F-fluorodeoxyglucose Positron Emission Tomography/Computed Tomography in a Patient with Lung Cancer

**DOI:** 10.7759/cureus.1124

**Published:** 2017-03-29

**Authors:** Daniele Penna, Natale Quartuccio, Claudio Testa, Vincenzo Arena, Angelina Cistaro, Ettore Pelosi

**Affiliations:** 1 PET/CT center, Affidea IRMET - Turin - Italy; 2 Wolfson Molecular Imaging Centre, University of Manchester

**Keywords:** fdg-pet, hibernoma, brown fat tissue, fat tissue tumor, liposarcoma, pet, lipoma

## Abstract

Hibernoma is a benign tumor arising from brown fat tissue. Conventional imaging techniques are not able to differentiate it from other benign lesions or malignant fatty tumors. We report the case of a 73-year-old patient who underwent a thorax computed tomography (CT) and was then referred to our department for metabolic assessment of a solitary lung nodule. An F18-fluorodeoxyglucose positron emission tomography/computed tomography (18F-FDG-PET/CT) scan was performed and demonstrated, in addition, a highly metabolic fat-containing lesion mimicking a malignant fatty tumor in the left great pectoralis muscle. The lesion was excised and resulted to be a hibernoma. This case shows that hibernoma can appear as a malignant-like lesion on 18F-FDG-PET/CT scan as per other imaging techniques, and the grade of FDG uptake does not accurately reflect malignancy in this fat-containing tumor. However, 18F-FDG-PET/CT with its whole-body scanning capability may represent a useful imaging tool in identifying, in the course of an imaging study for oncological evaluation, additional incidental findings such as benign fat-containing lesions that may require a surgical approach.

## Introduction

Hibernoma is a rare benign tumor originating from brown fat tissue of adult patients that usually develops in the subcutaneous tissue or, less frequently, into the muscle. The main location of this neoplasia is the thigh, but it has also been found at the level of the shoulder, axilla, mediastinum, and superior thorax [1-2]. Typically the tumor occurs in people aged between 30 and 50 years [2]. In the evaluation of this type of lesion, the traditional diagnostic techniques (including computed tomography (CT) and magnetic resonance imaging (MRI) are not able to distinguish with certainty between benign tumor, such as hibernoma or lipoma, and malignant tumor, such as liposarcoma [3]. Therefore after the identification of a potential neoplasia with fat tissue characteristics, further diagnostic investigations are necessary [4-7]. On the suspicion of a hibernoma, considering the high vascularization, a core needle biopsy is not recommended due to the risk of massive hemorrhage. Instead, with a radical surgical approach, the risk of bleeding is avoided and the lesion does not show the tendency to relapse [1-2, 4, 8-10]. Here, we present a case of hibernoma incidentally identified in the course of an 18F-fluorodeoxyglucose positron emission tomography/computed tomography (18F-FDG-PET/CT) examination.

## Case presentation

A 73-year-old female patient was referred to our centre to undergo a whole-body 18F-FDG-PET/CT scan in order to characterize a solitary lung nodule. The patient had previously undergone (a month ago) a thorax CT scan which detected a subpleural, slightly spiculated pulmonary nodule measuring approximately 2.5 cm in the left lower lobe.

The patient presented to our centre with a long-lasting cough and no other symptoms. At the time of the scan, the patient was not taking any medicine. The PET/CT scan was performed from the base of the skull to the proximal thighs on an integrated PET/CT scanner (Discovery ST-E, General Electric Medical Systems, Milwaukee, WI), approximately one hour after the intravenous administration of 18F-FDG (300 MBq) with the patient fasting for eight hours before the injection. PET data were acquired in 3-D mode on a matrix of 128 × 128 pixels. Low-dose CT (tube voltage = 80 kVp, tube current = 60 mA, pitch of helical = 3.75:1) was performed for attenuation correction and anatomical localization of the PET signal, and images were acquired on a matrix of 512 × 512 pixels.

**Figure 1:**
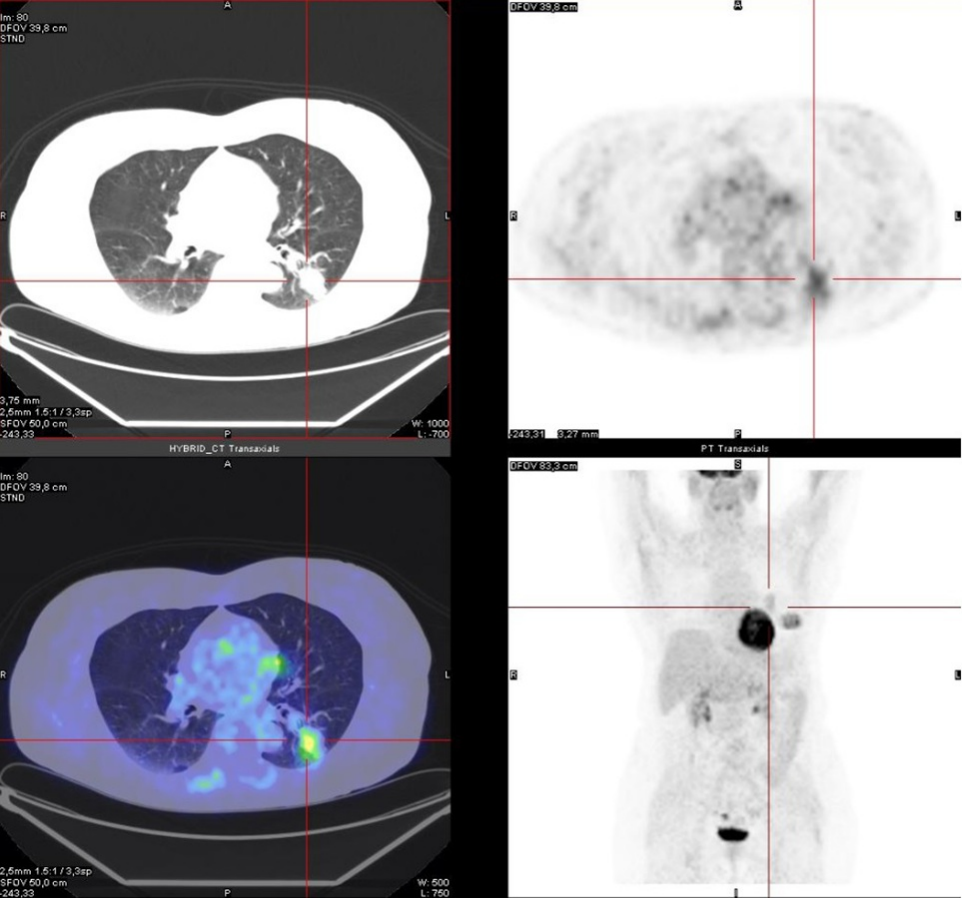
PET/CT image: pathological uptake of FDG at the left lung (SUV max 3.5). Histological examination led to the diagnosis of primary lung adenocarcinoma. FDG: Fluorodeoxyglucose; PET/CT: Positron emission tomography/computed tomography; SUV: Standardized uptake value.

**Figure 2:**
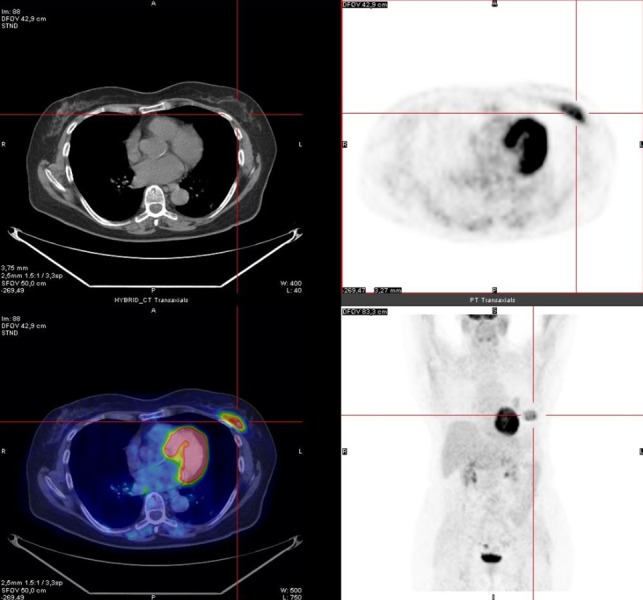
PET/CT image: abnormal uptake of FDG at the great left pectoral muscle (SUV max 5.8). Histological examination led to the diagnosis of hibernoma. FDG: Fluorodeoxyglucose; PET/CT: Positron emission tomography/computed tomography; SUV: Standardized uptake value.

The PET images demonstrated high FDG uptake in the known left pulmonary nodule with a maximum standardized uptake value (SUV max) of 3.5 (Figure 1). In addition, the PET/CT scan incidentally showed a large well-defined intramuscular FDG-avid mass (SUV max 5.8), with heterogenous low-attenuation on CT images in the context of the left great pectoral muscle (Figure 2) suggesting the presence of a tumor of the adipose tissue. Local lymph nodal stations were not FDG-avid and no other site of abnormal FDG uptake was detected.

Under the high suspicion of lung malignancy, the patient was referred for a core needle biopsy, confirming the presence of pulmonary adenocarcinoma, and subsequent left lower lobectomy.

Due to the malignant-looking fat-containing lesion, the patient also underwent excisional biopsy of the FDG-avid mass at the level of the left great pectoral muscle. Histological examination of the mass (hematoxylin and eosin stain) demonstrated multivacuolated eosinophilic fatty cells, the presence of skeletal muscle fibers and a highly vascular pattern in keeping with a diagnosis of hibernoma, and a rare benign tumor of the adipose tissue. The patient was referred for a first follow-up physical examination two weeks after the operation and then up to six months later, and no abnormal mass was identified on the site of the excision indicating no recurrence of the lesion.

## Discussion

Hibernoma is a slow-growing and painless lesion which can grow either in the subcutaneous tissue or with muscle. Due to the lack of specific symptoms, it is not rare to detect the lesion as an incidental finding [1-3]. Imaging features of MRI are not characteristic with predominantly hypointense or isointense signal in the fatty tissue on T2-weighted images [3]. On CT scan hibernoma can appear as a low-attenuation mass with diffuse enhancement [2].

There are few cases of hibernoma evaluated by 18F-FDG-PET/CT scan in the literature [6-7]. Hibernoma has been described as a hypermetabolic lesion on 18F-FDG-PET/CT examination [7]. The high metabolism is thought to be related to a large number of mitochondria within the brown adipocytes that present a high metabolic activity [6-9]. Differential diagnosis of benign tumors is complex and includes lipoma, hemangioma, and angiolipoma [10]. Similarly, differential diagnosis of malignant fatty tumors, such as liposarcoma, is not reliably achievable on the basis of the FDG uptake, although most liposarcomas may show low FDG metabolism [6-9]. On the other hand, core needle biopsy is not recommended as hibernoma has a very rich vascularisation and there is a risk of massive hemorrhage [1-2]. Kim and Lee previously reported on a case of hibernoma in a 43-year-old patient presenting a bulky mass in the subcutaneous layer of the left back. Interestingly, the lesion showed an increasing FDG SUV max over time (12.83 in the 1-h image and 20.83 in 2-h acquisition) [6].

In our case, as in the case reported by Kim and Lee, 18F-FDG-PET/CT was able to depict this fatty tumor [6]. In our patient, the 18F-FDG-PET/CT scan resulted to be useful due to the whole-body coverage and incidentally identified the hibernoma, as a highly metabolic lesion. Detection of this lesion is important, as the expanding mass, although benign, may compress neighboring anatomical regions and deserves surgical excision [2]. Although in the reported case the SUV max was intermediate, liposarcoma may also present similar FDG metabolism; therefore, SUV max cannot serve to distinguish between benign and malignant fatty lesions [7].

Our case is particularly interesting as it evidences the long-lasting evolution of this benign fatty neoplasm. At the time of the PET/CT scan, the patient was 73 years old, whereas the majority of the cases in literature have been described in patients who are in their 30s/40s [1-7].

## Conclusions

Our case indicates that SUV max may not accurately reflect the potential malignancy of fat tissue tumors. Hibernoma, although benign, may be mistaken for highly metabolic malignancy. However, 18F-FDG-PET/CT scan represents a valuable tool to identify benign expansive lesions of adipose tissue that could need a surgical approach.
